# Alcoholism and Immobility Induced Rhabdomyolysis Culminating in Hemodialysis

**DOI:** 10.7759/cureus.59316

**Published:** 2024-04-29

**Authors:** Sai Rakshith Gaddameedi, Phani Bhavana Cherukuri, Mahrukh A Khan, Suryansh Atreya, Vandana Bandari, Manjula Ashok, Shazia Shah

**Affiliations:** 1 Internal Medicine, Rutgers Health/Monmouth Medical Center, Long Branch, USA; 2 Internal Medicine, Bayhealth Medical Center, Dover, USA; 3 Nephrology, Rutgers Health/Monmouth Medical center, Long Branch, USA

**Keywords:** acute kidney injury (aki), continuous renal replacement therapy (crrt), creatine kinase, kidney injury, myoglobin, alcoholism, hemodialysis, rhabdomyolysis

## Abstract

Rhabdomyolysis is characterised by muscle breakdown and the release of myoglobin. It is a potentially serious condition that can lead to acute kidney injury (AKI). Factors, such as ischemia, trauma, muscle compression and drug toxicity, can trigger muscle breakdown. Treatment involves aggressive fluid resuscitation to maintain urine output and prevent renal injury. Severe cases with AKI may require temporary renal replacement therapy, such as haemodialysis. It has also been proposed that dialysis can speed up recovery by removing myoglobin that is secreted into the circulation by injured muscles. We present a case of a patient with alcohol abuse and prolonged immobility leading to severe rhabdomyolysis requiring hemodialysis. Our aim is to emphasise the importance of timely identification, and appropriate management of severe rhabdomyolysis not improving on fluids may require HD as soon as possible in order to minimise complications.

## Introduction

Rhabdomyolysis, characterised by muscle breakdown and the release of myoglobin, is a potentially serious condition that can lead to acute kidney injury (AKI). Rhabdomyolysis can arise from either traumatic or nontraumatic origins. Traumatic cases often result from crush injuries during accidents or disasters. Nontraumatic instances frequently stem from seizures, drug usage, dehydration, sepsis, alcohol, prolonged periods of immobility, and medication-causing acute renal failure. Alcoholism and prolonged immobility can contribute to skeletal muscle myopathy, making individuals more susceptible to rhabdomyolysis [1]. Rhabdomyolysis leads to the release of myoglobin, creatine kinase (CK), and electrolytes into the bloodstream, with myoglobin potentially causing kidney damage when it accumulates in renal tubules. Early recognition and management of rhabdomyolysis are crucial to prevent complications. Treatment involves managing the etiology of rhabdomyolysis and aggressive fluid resuscitation to maintain urine output and prevent renal injury. Severe cases with AKI may require temporary renal replacement therapy, such as hemodialysis. Hemodialysis is a supportive treatment that helps in removing myoglobin. Some removal of myoglobin has been seen with the use of high-cut-off dialysers or the use of hemodiafiltration.

In this case report, we describe the clinical presentation, diagnostic evaluation and management of a patient with a history of alcoholism and prolonged immobility who presented with rhabdomyolysis. The patient subsequently developed severe AKI necessitating temporary hemodialysis. This case report underscores the importance of heightened awareness of rhabdomyolysis in patients with predisposing factors, highlighting the challenges encountered in its management.

## Case presentation

A 55-year-old male with a medical history significant for alcohol use disorder presented to the ED for evaluation of severe lower back and thigh pain for two days. His pain was 10/10 in intensity, localised on the left lower back and the right thigh. It was achy, non-radiating, continuous pain, not relieved with painkillers. It was exacerbated by movement; therefore, he had difficulty getting out of bed and could not go to work, which has progressively worsened. He was lying in bed for four days, and his last alcohol intake was two days ago. He denied fevers, chills, abdominal pain, chest pain, falls, dizziness, statin use, cocaine use, seizure disorder or a recent history of trauma but reported that his urine was dark in colour. On physical exam, he was in mild distress due to pain and had dry buccal mucosa, abdominal distension, mild tenderness in the right upper and lower quadrants, severe left paraspinal and costovertebral angle (CVA) tenderness.

**Figure 1:**
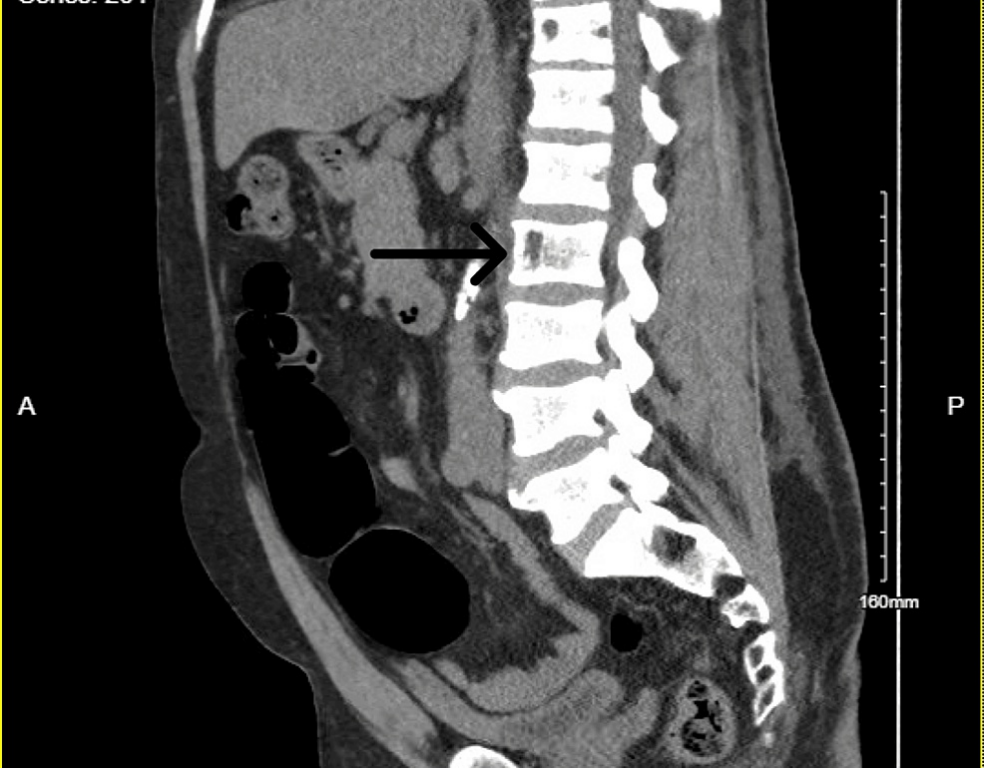
CT abdomen and pelvis illustrating several nonspecific osteolytic lesions noted in the thoracolumbar spine centred at L2.

**Figure 2:**
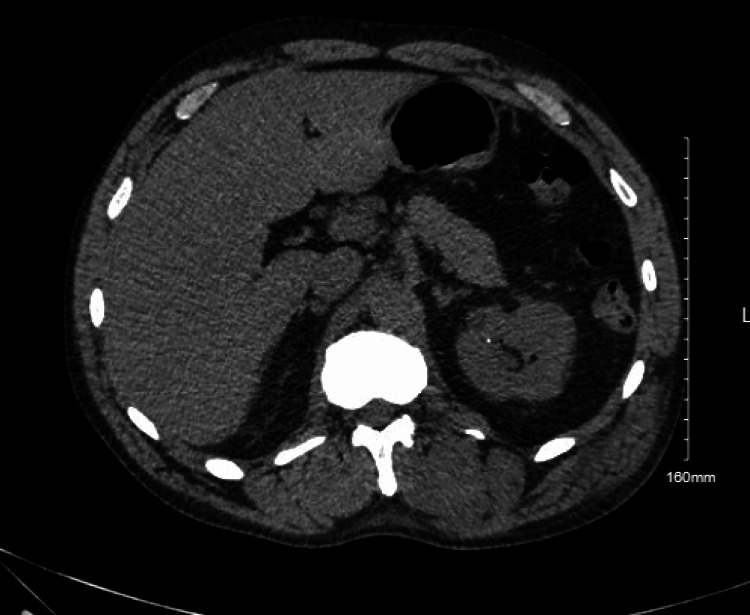
CT abdomen and pelvis demonstrating atrophy and cortical scarring involving the left kidney.

**Figure 3:**
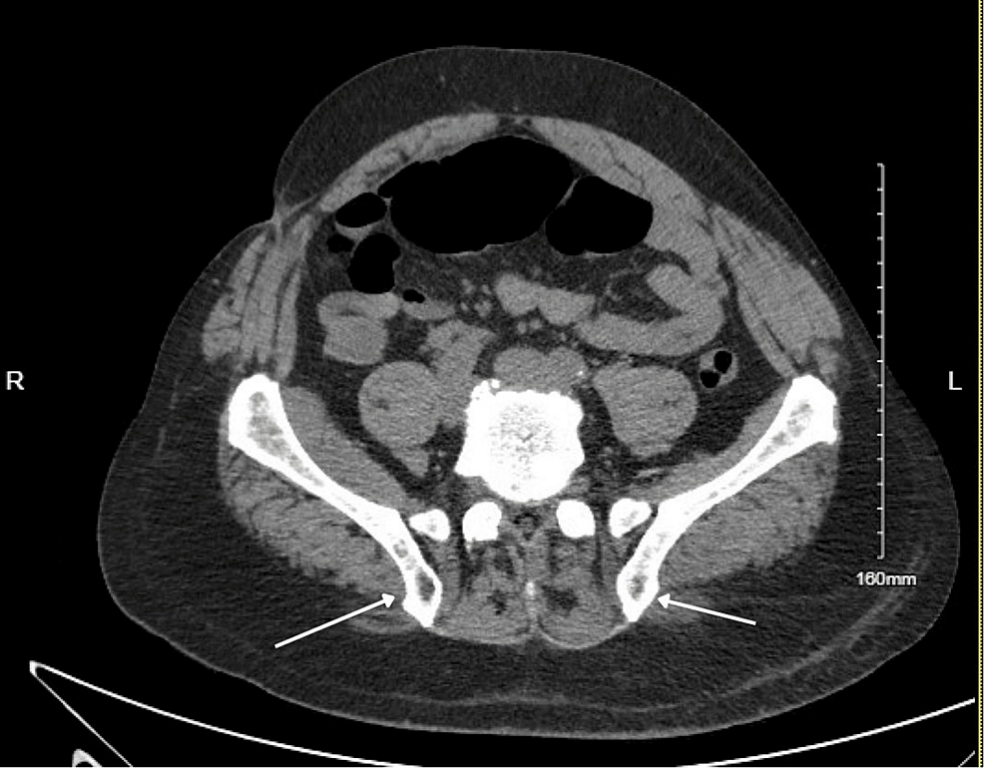
CT abdomen and pelvis illustrating several nonspecific osteolytic lesions noted in the bilateral iliac bones.

**Figure 4:**
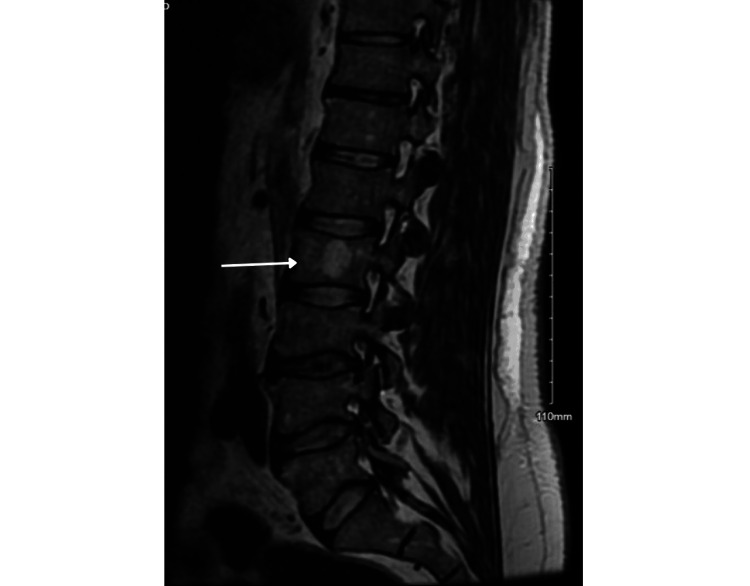
MRI lumbar spine demonstrating T1 and T2 intense lesions in the L2 likely representing intraosseous hemangioma.

In the ED, his BP was 161/75 mmHg, his heart rate was 117 bpm and his respiratory rate was 20 breaths per minute, with a temperature of 101 degrees Fahrenheit. On admission, labs were remarkable for leukocytosis with white blood cell (WBC) count of 11,800, haemoglobin 14 mg/dl, creatinine (Cr) 5.87 mg/dl (baseline Cr was 1 mg/dl), blood urea nitrogen (BUN) 43 mg/dl, aspartate transaminase (AST) 4916 U/L, alanine transaminase (ALT) 2827 U/L, alkaline phosphatase 141U/L, CK 33,394 U/L, ferritin 767 ng/ml, erythrocyte sedimentation rate (ESR) 88 mm/hr and C-reactive protein (CRP) 184 mg/dl. His platelets, international normalised ratio (INR) and bilirubin levels were in the normal range. Urine toxicology was negative, and blood alcohol level was less than 10 mg/dl. His urinalysis revealed proteinuria 300 mg/dl, specific gravity greater than 1.03, and blood, but red blood cells (RBC) and WBC were in normal ranges. CT abdomen and pelvis (Figures 1, 2, 3) without contrast revealed several osteolytic lesions noted throughout the spine and pelvis (likely believed to be due to intraosseus hemangiomas), chronic atrophy and scarring involving the left kidney, grossly normal right kidney and liver steatosis. MRI of the thoracic and lumbar spine (Figure 4) only revealed intraosseous hemangiomas.

A bone scan, hypercoagulable workup and myeloma workup were ordered, and the results returned normal. The patient received 2 litres of normal saline bolus and was started on 200 cc/hr normal saline continuous fluids for rhabdomyolysis. Over the next few days, his oliguria worsened to anuria with no response to fluids. His creatinine worsened from 5.87 mg/dl to 7.14 mg/dl, and potassium increased from 4.5 mEq/L to 5.3 mEq/L. Nephrology was consulted, and the decision was made to start the patient on emergent hemodialysis (HD). Urine output slowly improved over the next few days, along with improvement in creatinine and CK. He received three sessions of HD, after which his CK, Cr and LFTs were downtrending.

On the day of discharge, his CK level had come down to 776 U/L, and his creatinine had improved to 1.63 mg/dl. In the next few days, his creatinine normalised, and the decision was made to discharge the patient.

## Discussion

Rhabdomyolysis, derived from the Greek words rhabdos (meaning 'rod') + mus (meaning 'muscle') + lusis (meaning 'loosening'), describes the quick disintegration of skeletal (striated) muscle, releasing myoglobin into the circulation and potentially causing renal failure [2]. It is characterised by a rapid increase in the serum CK level and the development of either localised or generalised myalgia and weakness. The degree of this increase depends on when the analysis is done in relation to the acute event [3]. Alcohol and narcotics are among the many substances that can induce rhabdomyopathy. Alcoholic myopathy is categorised as acute, subacute or chronic and can appear in a variety of ways. Excessive alcohol use is linked to acute or subacute alcoholic myopathy, which is often characterised by localised or widespread pain and cramping in the muscles. Muscle biopsy specimens usually exhibit an ambiguous pattern with muscle fibre degradation and regeneration and high serum CK activity. The hallmark of chronic alcoholic myopathy is proximal muscular weakness, as observed in our patient, along with mild nonspecific changes evident in muscle biopsy specimens. Even in healthy individuals with a wholesome diet enhanced with vitamins, three weeks of daily use of 225 g of alcohol resulted in mild increases in serum CK activity and subtle but noticeable anomalies in muscle [4].

Although the precise mechanism by which rhabdomyolysis reduces glomerular filtration rate is unknown, there is mounting evidence that the following three factors may be to blame: 1) renal vasoconstriction, 2) direct and ischemic tubule injury, and 3) tubular blockage. When CK levels are below 15,000 to 20,000 U per litre upon admission, the likelihood of AKI in rhabdomyolysis is typically low [5-8]. However, our patient presented with a CK level of 33,394 U/L. CK levels as low as 5000 U per litre have been linked to AKI. However, this typically happens in conjunction with other diseases, such as sepsis, dehydration and acidosis.

The primary strategy and most common strategy for preventing and treating AKI is generally accepted to be early and vigorous fluid resuscitation to restore renal perfusion and enhance the urine flow rate. Patients with rhabdomyolysis may have up to 12 L of fluid trapped in their necrotic muscle tissues, which can lead to hypovolemia and, eventually, renal failure [9]. Saline solutions to expand/restore intravascular volume and therapy of the underlying cause of rhabdomyolysis are the cornerstones of attempts to prevent AKI in patients with rhabdomyolysis. Although early and aggressive volume resuscitation with the goal of boosting urine flow (between 200 and 300 mL/h) is generally accepted, there is no information regarding the appropriate fluids to deliver. While most cases resolve after aggressive fluid therapy, there are a few outliers who do not respond. They may require more invasive interventions like renal replacement therapy (which includes hemodialysis, hemodiafiltration, continuous renal replacement therapy and continuous veno-venous hemodialysis). Hemodialysis aids in the removal of some myoglobin, providing supportive treatment while addressing the underlying causes of rhabdomyolysis. One of the most common of these is anuric acute kidney injury characterised more precisely by an increase in serum creatinine by two to four times baseline or >4.0 mg/dl or urine output <0.3-0.5 ml/kg/d and with failure of diuresis following hyperhydration.

As our case was anuric, according to the guidelines laid down by KDIGO, he fulfilled the criteria for renal replacement therapy (hemodialysis in this instance). Other significant indications that require renal replacement include hyperkalemia, hypercalcemia, hyperazotemia and volume overload. Moreover, the literature, in general, suggests that hemodiafiltration or continuous renal replacement therapy (CRRT) may be more effective in treating rhabdomyolysis refractory to conventional treatment as myoglobin is a large molecule that may be removed easily by convective transport (e.g. CRRT) rather than diffusive transport (e.g. ischemic heart disease (IHD)) [10].

## Conclusions

We therefore suggest using invasive strategies in treating rapidly worsening rhabdomyolysis fulfilling the criteria. One must not shy away from using hemodialysis if other more specialised renal replacement options (CRRT and CVVHD) are not available in order to prevent aggravation of complications. It may also be noteworthy to keep in mind that we treat the manifestations of acute kidney injury as a result of myoglobin toxicity and ensuing major complications irrespective of the actual CK levels.

Prompt recognition of rhabdomyolysis in chronic alcoholics presenting with myopathy symptoms is crucial. Early aggressive resuscitation, coupled with prompt identification of the need for renal replacement therapy in those resistant to fluid resuscitation, is essential for optimal patient outcomes.
